# Efficacy and safety of robot-assisted vs. stereotactic framework deep brain stimulation for Parkinson’s disease: a retrospective study

**DOI:** 10.3389/fnagi.2026.1699583

**Published:** 2026-02-27

**Authors:** Lian Meng, Xiaojian Wang, Xinyi Liang, Zhenhua Mo, Weiming Liang, Jieru Quan, Zhengde Xie, Jinyu Huang

**Affiliations:** 1 The First Affiliated Hospital of Guangxi University of Science and Technology, Guangxi University of Science and Technology, Liuzhou, Guangxi Province, China; 2 School of Economics and Management, Guangxi University of Science and Technology, Liuzhou, Guangxi Province, China

**Keywords:** deep brain stimulation, electrode implantation accuracy, levodopa equivalent dose, Parkinson’s disease, robot-assisted, unified Parkinson’s disease rating scale

## Abstract

**Background:**

This retrospective study aimed to compare the efficacy and safety of robot-assisted deep brain stimulation (DBS) vs. stereotactic framework DBS for Parkinson’s disease (PD).

**Methods:**

The present study constituted a retrospective analysis that integrated a review of medical records with an outcomes management database from the First Affiliated Hospital of Guangxi University of Science and Technology. A total of 32 patients experienced robot-assisted DBS with the Sinovation surgical robot system, while an additional 30 patients underwent stereotactic frame DBS. The primary objective was the accuracy of electrode implantation, whereas the secondary objectives were postoperative UPDRS-III scores, levodopa equivalent dose (LEDD), and complications.

**Results:**

No substantial difference was observed between the two groups concerning sex, age, disease duration, Hoehn-Yahr score, preoperative UPDRS-III scores, and preoperative LEDD. In comparison to stereotactic framework DBS, robot-assisted DBS demonstrated a markedly reduced average targeting error (1.43 ± 0.51 mm vs. 1.91 ± 1.38 mm, *p* = 0.034). Moreover, robot-assisted DBS demonstrated a markedly greater reduction in UPDRS-III scores and a decrease in LEDD at 3, 6, and 12 months postoperatively. No fatalities or lasting complications due to surgery were detected in either group during the study period.

**Conclusion:**

In comparison to stereotactic framework DBS, robot-assisted DBS demonstrated notable benefits in electrode implantation precision, UPDRS-III scores, and LEDD. Robot-assisted deep brain stimulation with the Sinovation surgical robot system was recommended for the management of Parkinson’s disease.

## 1 Introduction

Parkinson’s disease (PD) ranks as the second most prevalent neurodegenerative disease globally. It is a progressively advancing disorder caused by the depletion of dopaminergic neurons in the substantia nigra and the deterioration of their projections to the striatum (caudate nucleus and putamen; GBD 2016 Parkinson's Disease Collaborators, 2018; Lees et al., 2009; Lindvall, 2016). According to the Global Burden of Disease (GBD) findings, neurological disorders are the foremost contributors to the global disability burden, with Parkinson’s disease demonstrating the highest growth rate among these conditions based on age-standardized prevalence, disability-adjusted life years (DALYs), and mortality statistics (Dorsey et al., 2018). WHO forecasts indicate that neurodegenerative illnesses, including PD and Alzheimer’s disease (AD), will become the second greatest cause of worldwide mortality by 2040, exceeding cancer-related fatalities (Gammon, 2014). Parkinson’s disease is marked by low mortality but significant impairment, primarily seen as a progressive decline in voluntary motor function. This fundamental clinical characteristic has a consistently growing incidence with advancing age (Frisardi et al., 2016). Dopaminergic replacement therapy based on levodopa has been the gold standard for more than 50 years, however its effectiveness gradually declines as the disease progresses. Chronic administration of levodopa often leads to medication-induced dyskinesias and motor fluctuations, hence worsening disability and placing significant socioeconomic difficulties on caregivers (Garcia-Ruiz et al., 2014).

In 1987, a research team headed by French neurosurgeon Alim-Louis Benabid initiated the clinical implementation of DBS, signifying a groundbreaking advancement in functional neurosurgery (Benabid et al., 1988). Thalamic DBS technology aimed at the thalamus has obtained worldwide regulatory approval, including authorization from the Food and Drug Administration (FDA) and CE Mark certification. This intervention is currently recommended for Parkinson’s disease patients with medically resistant responses to levodopa medication and has increasingly replaced thalamotomy for the treatment of parkinsonian tremor and essential tremor (Rehncrona et al., 2003; Okun, 2012). DBS is internationally recognized as the primary method for addressing motor symptoms in advanced-stage Parkinson’s disease. This neurosurgical technique involves the initial stereotactic localization of target nuclei, followed by the implantation of stimulating microelectrodes in structures such as the subthalamic nucleus (STN) or globus pallidus internus (GPI), thereby modulating neuronal excitability to alleviate symptoms of Parkinson’s disease (Rodriguez et al., 2007). The accumulation of clinical experience using subthalamic nucleus DBS for severe PD has coincided with its increasing acceptance among neurologists, neurosurgeons, and patients. This convergence has heightened emphasis on enhancing surgical targeting accuracy to reduce procedure-related morbidity (Terao et al., 2003; Starr, 2002; Aziz et al., 2001; Bejjani et al., 2000).

Accurate targeting is essential, as the therapeutic advantages of DBS rely on the selective neuromodulation of certain brain regions, which is dependent on exact electrode placement (Bot et al., 2018; Li et al., 2016). Stereotactic frames are the benchmark for accurate electrode placement in functional neurosurgery, establishing the fundamental design model for modern neurosurgical robotic systems. This method possesses considerable constraints: 1. Cranial fixation requirements impose physical limitations; 2. Significant frame weight induces patient discomfort; 3. Psychological aversion exists in specific demographics; 4. Contraindicated in pediatric patients due to incomplete cranial development; 5. Visual field obstruction and limited operational space arise from frame complexity; 6. Installation processes are time-consuming (Spiegel et al., 1947; Benabid et al., 2003; Deuschl et al., 2003; Lanotte et al., 2002; Zhou et al., 2023).

The neurosurgical domain is experiencing the swift development of advanced robotic systems aimed at improving procedural precision in DBS, brain biopsies, and therapeutic ablations. After the first robotic application in neurosurgery in 1985, notable advancements include the NeuroMate system in 1987, followed by FDA approvals in 2012 for the ROSA Brain and Renaissance robotic systems for intracranial procedures (Faraji et al., 2020). A recent meta-analysis of 27 trials conducted over 16 years comparing DBS implantation procedures indicated a pooled mean aiming error of 1.91 mm across all modalities. Furthermore, robotic aid correlated with a mean decrease in targeting error of 0.79 mm (Philipp et al., 2021). Sinovation Medical is established as a prominent domestic producer of neurosurgery robotic systems in China. The Sinovation surgical robot system (SR1-3D) exemplify Chinese-developed neurosurgical robots, engineered with modular architectures tailored for stereotactic procedures such as DBS lead installation, biopsy sampling, and SEEG electrode implantation (Zhou et al., 2023; Li and Guo, 2021).

Currently, there is limited research assessing the efficacy and safety of Chinese-developed robotic systems in DBS. This retrospective study aimed to evaluate the efficacy and safety of the Sinovation surgical robot system-assisted DBS for Parkinson’s disease. The major objective was the precision of electrode implantation, whereas the secondary objectives were UPDRS-III scores, LEDD, surgical durations, and safety outcomes.

## 2 Materials and methods

### 2.1 Study design

This was a retrospective study that combined a review of medical records with an outcomes management database from the First Affiliated Hospital of Guangxi University of Science and Technology. The data for this database was collected from persons diagnosed with Parkinson’s disease who fulfilled the inclusion criteria between April 2022 and March 2024.

### 2.2 Ethical approval and consent

The study was executed in accordance with the Declaration of Helsinki. Approvals from the Ethics Committee of the First Affiliated Hospital of Guangxi University of Science and Technology were obtained in March 2025 (approval number: 2025-LC121).

### 2.3 Inclusion and exclusion criteria

The criteria for inclusion were as follows:(1) Patients with clinically confirmed PD and disease duration ≥5 years (age restrictions may be removed for DBS candidates whose symptoms severely impair quality of life despite meeting all other surgical indications); (2) Patients historically demonstrating significant clinical response to levodopa/carbidopa but currently exhibiting discernible “wearing-off” phenomena and motor fluctuations, or patients exhibiting intolerable medication-related adverse effects that compromise therapeutic efficacy; (3) A positive acute levodopa challenge test—demonstrating ≥30% improvement in MDS-UPDRS Part III motor scores—served; (4) Hoehn-Yahr stages 2.5 to 5; (5) Patients exhibiting pharmacologically refractory tremor, rigidity, or non-motor manifestations.

The criteria for exclusion were as follows: (1) Patients with dementia or clinically significant cognitive impairment. (MMSE Scale <17). (2) Patients with severe depressive states, anxiety disorders, or schizophrenia (HAMD≥24). (3) Patients with uncontrolled comorbidities—including severe hypertension, diabetes, or cardiac diseases. (4) significant cerebral atrophy; cerebrovascular pathology; intracranial abnormalities; or history of craniovertebral surgery within 6 months.

### 2.4 Preoperative preparation

#### 2.4.1 Stereotactic framework DBS

The patient had a 3.0 T high-resolution MR scan during the preoperative medication phase to alleviate discomfort during the procedure. If the patient was uncooperative, a minimal dosage of sedative medication would be administered. The scanning range spanned from the base to the apex of the skull, with T1, T2, SW and susceptibility sequences. The image was scanned and uploaded into the Framelink 5.0 planning system. Lidocaine was administered in the ward. Lekseller-g stereotactic frames and imaging plates were positioned under local infiltration anesthesia according to the designated sites. The positioning frame was affixed to the external surface of the skull via screws. The Y-axis of the base ring of the frame must be parallel to the projection line of the AC-PC body surface, and the midpoint of the transverse column at the front of the frame should align with the median sagittal plane of the patient. The stereotactic frame was secured to the CT adaptor, maintaining a perpendicular orientation between the frame and the scanning bed. The setting for CT scanning was fundamentally identical to that of MRI, utilizing a flat scan from the base of the skull to the apex of the scalp. The imaging data was integrated into the surgical planning system. In the Framelink surgical planning system, CT scans served as the foundation for the fusion of magnetic resonance T1 and T2 imaging with CT, respectively. In accordance with the T1 sequence, AC and PC points were delineated on the axial, coronal, and sagittal images, respectively. The target location was established on the T2 sequence, and the needle insertion pathway was simulated. The needle tract design circumvents the sulci, lateral ventricle, and visible blood vessels on imaging to mitigate the risks of bleeding and electrode displacement, given the high density of blood vessels in the sulci and the potential for electrode migration within the lateral ventricle.

#### 2.4.2 Robot-assisted DBS

Prior to the procedure, install the foundational frame of the Sinovation surgical robot. Thin-layer CT scanning was conducted encompassing the entire cranium and the foundation frame from the inferior aspect of the skull to the superior aspect. The CT images [CT threshold: brain window (150, 30), bone window (800, 400)] and various MRI sequences (T1WI, T2WI) were imported into the Sinoplan surgical planning system. We integrated and cataloged the pertinent imaging data, reconstructed the three-dimensional image, established the anterior commissure (AC) and posterior commissure (PC) coordinate system, designated the anterior commissure position as AC and the posterior commissure position as PC in the axial view, and randomly selected a point superior to AC and PC in the sagittal view as IH. In the T2 image, we identified the largest cross-section of the red nucleus, drew a parallel line from the upper edge of this layer, and designated the intersection point with the STN at the inner third as the target point. The T1 image was utilized to modify and circumvent sulci, gyri, and blood vessels. We configured the ARC and RING, and established the entrance point into the skull. Then the surgical path design was finalized.

### 2.5 Surgical procedure

The choice of surgical approach was based on the availability of the Sinovation surgical robot. The Sinovation surgical robot was leased and could not be guaranteed to be available at any time. When the robot was available, robot-assisted DBS was performed; otherwise, stereotactic frame-based DBS was performed. Both robot-assisted DBS and stereotactic frame-based DBS were performed by the same team of experienced surgeons.

#### 2.5.1 Stereotactic framework DBS

Upon entering the operating room, the patient assumed a supine position. The Leksell G-frame stereotactic system (Elekta AB, Stockholm, Sweden) was fixed on the operating table. General anesthesia was administered, followed by standard decontamination and dressing procedures. The X, Y, Z, Ring, and Arc values of the stereotactic frame were calibrated in accordance with the preoperative plan, and the coordinates were verified by two individuals. A sterile probe protection sleeve was fitted into the arch frame guide slot to safeguard the cannula, indicating the entry location into the skin. A curved or longitudinal incision was executed, penetrating the skin, followed by meticulous hemostasis, after which a cranial bone drill was utilized, and the bone plate was occluded with bone wax. The base ring affixed to the DBS electrode was secured to the bone aperture. The identical technique was employed to incise the contralateral scalp and perforate the aperture for redundancy. Upon verifying the precision of the plan coordinates, the dura mater was incised, the probe needle was introduced, and the osseous aperture was occluded with fibrin adhesive. A test microelectrode was introduced. The microelectrode typically initiates signal monitoring from 10 mm above the target location until the substantia nigra signal was detected. The insertion site was determined by selecting the depth of the needle path, which must be at least 3 mm, traversing the STN or GPI, and exhibiting the optimal nucleus signal. The electrode (type 3,389, Medtronic Inc., Minneapolis, MN) was implanted and linked to the Medtronic 8,840 programmer for macro-stimulation during surgery, with subsequent observation of the patient’s symptom improvement and side effects. The identical procedure was employed for the contralateral surgery and evaluation. Upon completion of both surgical sides, the stereotactic frame was extracted, general anesthesia was reinstated, and cleaning and dressing were reapplied prior to the implantation of the pulse emitter.

#### 2.5.2 Robot-assisted DBS

After entering the operating room, the Sinovation surgical robot (Sinovation Medical Technology Co, Ltd., Beijing, China) was positioned on the right side. The Y-shaped support was installed and connected directly to the robotic arm through the Y-shaped support. The patient’s head was positioned directly below the robotic arm, and the connection rod was locked to fix the robotic arm. Upon finalizing the surgical plan, we imported the preoperative CT images, executed fusion and registration to delineate the surface, chose three points as the preoperative registration markers, and maintained the registration error within 0.5, ensuring the error value remains acceptable. Upon registration, we performed standard cleaning and dressing procedures, and confirmed precision utilizing the base frame reference points. Then, we designed the surgical incision and the electrode entry place on the scalp under the direction of the surgical robot. An arc or longitudinal incision was performed to reveal the skull, then the drilling locations were identified on the skull based on the positioning of the mechanical arm. Following the successful drilling of the skull, we executed secondary registration and verified the target points. Upon connecting the microelectrode recording system, we incised the dura mater, inserted the cannula needle, introduced the electrophysiological needle, and performed intraoperative electrophysiological monitoring. Deep electrodes (type 3,389, Medtronic Inc., Minneapolis, MN) were implanted for intraoperative evaluation. The testing content encompassed the threshold for symptom amelioration and the threshold for adverse responses. Upon obtaining appropriate test results, we secured the electrodes and temporarily implanted the deep electrodes subcutaneously. The frame was extracted, general anesthesia was reinstated, and the nerve stimulator was inserted after reapplying disinfectant and dressing.

### 2.6 Follow-up and observation indicators

On the first day post-awakening and 1 week following the surgery, thin-layer CT scans were performed to assess the precise implantation position of the electrodes, and any complication. The postoperative CT scans were recorded onto disks and sent to the computer workstation for multimodal image fusion. The variance in distance between the electrode tip position and the designated target point in three-dimensional space was computed, and the mean value was established. By using the 3D Euclidean distance measurement algorithm ( d=√[(X−X0)^2+(Y−Y0)^2+(Z−Z0)^2] ), the straight-line distance between the designed electrode target point and the actual electrode implantation target point in the spatial domain was calculated.

Postoperative routine follow-up was conducted for the patients. The pulse generator was powered on and calibrated 1 month after the surgery. At 3, 6, and 12 months after the surgery, the patients were evaluated using the third part of the Parkinson’s Disease Rating Scale (MDS-UPDRS-III) and the LEED.

### 2.7 Statistical analysis

The Statistical Package for the Social Sciences (SPSS, version 24.0) was employed for the statistical analysis of the data. Numerical variables are represented as mean ± standard deviation. The Mann–Whitney U test was employed to assess the significance of differences between the two groups. Nominal variables were analyzed using Pearson’s chi-squared test. Differences were deemed significant at *p* < 0.05.

## 3 Results

### 3.1 Basic characteristics of patients

This study totally included 62 patients with PD undergoing DBS treatments at the First Affiliated Hospital of Guangxi University of Science and Technology from April 2022 to March 2024. Table 1 presented the basic characteristics of patients in the two groups. There was no significant difference between the two groups regarding sex, age, disease duration, Hoehn-Yahr score, preoperative UPDRS-III scores and preoperative LEDD. The surgical duration in the robot-assisted group was significantly shorter than that in the stereotactic framework group.

**Table 1:** Basic characteristics of patients.

Characteristic	Stereotactic framework group (*n* = 30)	Robot-assisted group (*n* = 32)	*p* value
Mean age (year)	55.2 ± 9.7	55.7 ± 9.5	0.82
Male	15	18	0.81
Disease duration (years)	7.7 ± 0.9	7.9 ± 0.9	0.36
Hoehn-Yahr score	4.2 ± 0.7	4.2 ± 0.7	0.95
preoperative UPDRS-III scores	34.2 ± 3.8	36.1 ± 4.2	0.06
preoperative LEDD (mg)	911 ± 138	913 ± 132	0.95
Average surgery time (min)	332 ± 18	259 ± 19	0.01

### 3.2 Electrode implantation accuracy

The average targeting error in the robot-assisted group was significantly lower than that in the stereotactic framework group (1.43 ± 0.51 mm vs. 1.91 ± 1.38 mm, *p* = 0.034; Table 2).

**Table 2:** Comparison of three-dimensional coordinate values between designed and actual targets (mm).

Outcome	Stereotactic framework group		Robot-assisted group		*p* value
Coordinate position	Designing target points	Actual target	Designing target points	Actual target	
x	11.89 ± 1.05	11.52 ± 1.12	12.61 ± 8.12	13.68 ± 3.88	
y	−1.57 ± 1.02	−2.18 ± 1.58	−1.52 ± 2.55	−1.95 ± 2.52	
z	−5.13 ± 0.97	−5.82 ± 1.72	5.48 ± 1.44	5.35 ± 1.51	
Average target error	1.91 ± 1.38		1.43 ± 0.51		0.034

### 3.3 Decrease of UPDRS-III scores

Compared with the stereotactic framework group, the decrease of UPDRS-III scores in the robot-assisted group was significantly higher at 3 months (11.3 ± 4.0 vs. 14.0 ± 4.5, *p* = 0.015), 6 months (13.2 ± 4.9 vs. 15.6 ± 4.4, *p* = 0.047), and 12 months (13.6 ± 5.1 vs. 16.8 ± 4.3, *p* = 0.007; Table 3).

**Table 3:** Reduction of UPDRS-III scores.

Outcome	Stereotactic framework group	Robot-assisted group	*p* value
Three months after the operation	11.3 ± 4.0	14.0 ± 4.5	0.015
Six months after the operation	13.2 ± 4.9	15.6 ± 4.4	0.047
Twelve months after the operation	13.6 ± 5.1	16.8 ± 4.3	0.007

### 3.4 Decrease of LEDD

Compared with the stereotactic framework group, the decrease of LEDD in the robot-assisted group was significantly higher at 3 months (185 ± 45 mg vs. 226 ± 50 mg, *p* < 0.001), 6 months (421 ± 53 mg vs. 497 ± 58 mg, *p* < 0.001), and 12 months (436 ± 54 mg vs. 536 ± 62 mg, *p* < 0.001; Table 4).

**Table 4:** Reduction of LEDD from preoperative levels at follow-up time points (x̄ ± s).

Outcome	Stereotactic framework group	Robot-assisted group	*p* value
Three months after the operation	185 ± 45 mg	226 ± 50 mg	<0.001
Six months after the operation	421 ± 53 mg	497 ± 58 mg	<0.001
Twelve months after the operation	436 ± 54 mg	536 ± 62 mg	<0.001

### 3.5 Safety outcomes

Each group experienced one instance of lung infection, and both instances resolved within the initial postoperative week following standardized treatment with nebulized expectorants and specific antibiotic therapy. One case in the stereotactic framework group suffered.

cerebral bleeding, and resolved spontaneously within 24–72 h without the need for surgical intervention. One case in the robot-assisted group suffered postoperative clouding of consciousness, with spontaneous clearance occurring within 24–48 h without pharmaceutical intervention.

## 4 Discussion

This retrospective study aimed to assess the efficacy and safety of robot-assisted vs. stereotactic framework DBS in 62 Parkinson’s disease patients treated at the First Affiliated Hospital of Guangxi University of Science and Technology from April 2022 to March 2024. The robot-assisted cohort exhibited markedly enhanced electrode implantation accuracy in comparison to the Leksell frame-assisted group (*p* < 0.05). Notwithstanding similar preoperative levels, the robot-assisted cohort exhibited significantly higher decrease in terms of UPDRS-III scores and LEDD compared with the stereotactic framework group at all postoperative follow-up intervals (*p* < 0.05). No surgery-related death or enduring complication was observed in either group throughout the study period.

The results of this study revealed that the mean targeting error in the robot-assisted group was considerably lower than that in the Leksell frame-assisted group. In contrast, the Leksell frame-assisted cohort demonstrated statistically significant targeting disparities across all three axes (X/Y/Z, *p* < 0.05), confirming that frame-based techniques do not attain equivalent precision levels. Benabid AL et al. discovered that a lead deviation above 2 mm from the targeted nucleus may diminish treatment efficacy. Numerous prior investigations indicated that the robot-assisted group showed markedly reduced standard deviations in vector error relative to the frame-based group, hence affirming enhanced targeting precision in electrode insertion (Postuma et al., 2015; Lozano et al., 2019; Fasano et al., 2012; Jha et al., 2017). Zhilong Huang et al. aggregated results from various studies, revealing that the vector error for robotic-assisted DBS surgery was 1.09 mm (95% confidence interval: 0.87 to 1.30) in patients with PD (Huang et al., 2024). The technological advantage was further validated by the significantly reduced percentage of vector errors over the 2 mm safety threshold in the robot-assisted group (10.7% vs. 34.2%, OR = 0.21, 95% CI: 0.09–0.45; Mei et al., 2022). Surgical robotic systems aim to reduce human errors and optimize surgical workflows, ultimately improving operational efficiency without compromising therapeutic efficacy. Stereotactic precision is essential for outcomes in movement disorders; nonetheless, it often proves to be laborious, susceptible to errors, and time-intensive to attain satisfactory levels (Deuschl et al., 2006; Starr et al., 2004). Surgical robots may adeptly maneuver around barriers and prevent harm to surrounding structures due to their accurate, consistent, and preordained trajectories, hence enhancing surgical precision. The reduced accuracy in frame-based systems may occur from deformation, wear, and blunting caused by extended thermal expansion of mechanical components and unintentional collisions. Robotic technologies possibly exhibit enhanced precision and accuracy in electrode implantation relative to frame-based methods; nevertheless, this requires consistent, comprehensive, and dependable maintenance practices (Mei et al., 2022). DBS robot-assisted surgery attains exceptional precision by the utilization of a reengineered registration procedure, intraoperative registration, and simulated target validation (Ribault et al., 2021; Gong et al., 2020; Jin et al., 2020).

Regarding UPDRS-III scores, the robot-assisted cohort exhibited significantly higher decrease in UPDRS-III scores from the stereotactic framework group at all postoperative follow-up intervals (*p* < 0.05). Multiple previous studies have shown markedly superior enhancements in UPDRS-III scores from preoperative baselines at 3, 6, and 12 months postoperatively in the robot-assisted group compared to the frame-based cohort (*p* < 0.05; Moriarty et al., 1996; Morrison et al., 2016; Mert et al., 2015). Previous investigations indicate varying enhancements in estimated UPDRS-III scores after DBS, likely linked to differences in stimulation frequency, programmed voltage settings, and drug dosages. Robot-assisted DBS has shown considerable superiority compared to traditional frame-based methods in improving UPDRS-III rates and treatment stability. This advancement was credited to the combined evolution of surgical precision and postoperative treatment, wherein submillimetric targeting accuracy guarantees exact electrode insertion inside the STN sensorimotor subregion. This establishes an anatomical basis for high-frequency stimulation to effectively mitigate abnormal beta oscillations, hence improving tremor control efficacy compared to the deficient stimulation paradigms typical of frame-based methods (Frequin et al., 2020). Moreover, the therapeutic benefit compared to frame-based cohorts demonstrated a progressively cumulative improvement over time.

Regarding LEDD, the robot-assisted group exhibited significantly greater decrease LEDD compared to the Leksell frame-assisted group. Similarly, multiple previous studies have reported much greater decreases in LEDD from preoperative values at postoperative months 3, 6, and 12 in the robot-assisted cohort compared to the frame-based group (Huang et al., 2024; Mei et al., 2022; Bezchlibnyk et al., 2021; Matias et al., 2018; Neudorfer et al., 2018; Chuang et al., 2023). This phenomenon indicated an advancement in therapeutic efficacy in robotic treatments, presumably facilitated by long-term optimization of neuromodulation by accurate electrode positioning. The fundamental mechanisms may encompass disparities in targeting precision (≤0.5 mm robotic error vs. ≥1.9 mm frame-based) and neuroplasticity windows—especially during the crucial 6–12 month postoperative phase when neural circuit remodeling transpires, wherein prolonged optimized stimulation may elicit cumulative neuromodulatory effects (Liu et al., 2017). This innovative method transforms previously documented correlations of stimulation parameters (frequency/amplitude/medication dosage) into measurable and reproducible therapeutic improvements (Mueller et al., 2018). Robot-assisted DBS demonstrated a substantial superiority in medication reduction starting at 3 months postoperatively, with a progressive enhancement in therapeutic effects observed over time.

Regarding safety outcomes, no surgery-related death or enduring complication was observed in either group throughout the study period. All complications in this study were temporary and completely reversible. Previous studies reported that DBS procedure may lead to complications such as subdural hematoma, cerebral infection, lung infection, intracranial hemorrhage, and postoperative altered consciousness (Lahtinen et al., 2020; Patel et al., 2015; Hu et al., 2010; Boviatsis et al., 2010; Doshi, 2011). Electrode stimulation may independently cause adverse effects, primarily presenting as pyramidal tract responses, apart from implant-related problems such as device rejection, poor wound healing, and lead migration (Li et al., 2017). The occurrence of pulmonary infection may be attributable to advanced age, compromised physiological reserve, and prolonged postoperative immobilization characteristic of Parkinsonian patients. Intracranial bleeding was hypothesized to be associated with gradual peri-electrode venous extravasation and previous comorbidities. The case of intracranial bleeding in this study demonstrated a positive clinical trajectory for surgical clouding of consciousness, with spontaneous clearance occurring within 24–48 h without the need for pharmaceutical treatment.

This study had several limitations. First, our study was a retrospective study. Data were derived from medical charts that may exhibit omissions, inaccuracies, or inconsistently documented information. The choice of surgical approach depended on the availability of the “Sinovation Surgical Robot,” which was a non-randomized, reality-based allocation method. This might introduce potential selection bias or time-related confounding factors. In the absence of randomization, baseline variables (e.g., age, comorbidities) might vary between groups, resulting in skewed comparisons. Second, patients in this study were selected from only one hospital, reducing generalizability. Finally, the sample size of the surgery was relatively small. Therefore, meticulously designed randomized controlled trials are essential for further validation of our findings in the future.

In conclusion, compared with stereotactic framework DBS, robot-assisted DBS exhibited significant advantages regarding electrode implantation accuracy, UPDRS-III scores and LEDD. Robot-assisted DBS using the Sinovation surgical robot system was advised for the treatment of Parkinson’s disease.

## Data Availability

The original contributions presented in the study are included in the article/supplementary material, further inquiries can be directed to the corresponding authors.
